# Establishing confidence in the output of qualitative research synthesis: the ConQual approach

**DOI:** 10.1186/1471-2288-14-108

**Published:** 2014-09-20

**Authors:** Zachary Munn, Kylie Porritt, Craig Lockwood, Edoardo Aromataris, Alan Pearson

**Affiliations:** The Joanna Briggs Institute, The University of Adelaide, Adelaide, 5005 South Australia

**Keywords:** Qualitative systematic reviews, Confidence, Credibility, Summary of findings, Meta-aggregation

## Abstract

**Background:**

The importance of findings derived from syntheses of qualitative research has been increasingly acknowledged. Findings that arise from qualitative syntheses inform questions of practice and policy in their own right and are commonly used to complement findings from quantitative research syntheses. The GRADE approach has been widely adopted by international organisations to rate the quality and confidence of the findings of quantitative systematic reviews. To date, there has been no widely accepted corresponding approach to assist health care professionals and policy makers in establishing confidence in the synthesised findings of qualitative systematic reviews.

**Methods:**

A methodological group was formed develop a process to assess the confidence in synthesised qualitative research findings and develop a Summary of Findings tables for meta-aggregative qualitative systematic reviews.

**Results:**

Dependability and credibility are two elements considered by the methodological group to influence the confidence of qualitative synthesised findings. A set of critical appraisal questions are proposed to establish dependability, whilst credibility can be ranked according to the goodness of fit between the author’s interpretation and the original data. By following the processes outlined in this article, an overall ranking can be assigned to rate the confidence of synthesised qualitative findings, a system we have labelled ConQual.

**Conclusions:**

The development and use of the ConQual approach will assist users of qualitative systematic reviews to establish confidence in the evidence produced in these types of reviews and can serve as a practical tool to assist in decision making.

## Background

Across the health professions, the impetus to practice evidence-based healthcare has increased exponentially since the term ‘Evidence-Based Medicine’ was first coined in 1992 [1]. Evidence-based practice has been defined as ‘…the conscientious, explicit, and judicious use of current best evidence in making decisions about the care of individual patients,…’ [2]. Rigorously conducted systematic reviews of the evidence are viewed as the pillar on which evidence-based healthcare rests and uniquely provide this foundation role by presenting health professionals with a comprehensive synthesis of the extant literature on a certain healthcare topic [2–4]. Evidence-based organisations such as the Cochrane Collaboration and the Joanna Briggs Institute (both established in the 1990s) were formed to develop methodologies and standards for the conduct of systematic reviews to inform decision making in healthcare [5–8]. Historically, systematic reviews have predominantly been undertaken to address questions regarding the effectiveness of interventions used in healthcare and therefore have required the analysis and synthesis of quantitative evidence. However, qualitative systematic reviews that bring together the findings of multiple, original qualitative studies, also have an important role in evidence-based healthcare. Qualitative systematic reviews can address questions to inform healthcare professionals about issues that cannot be answered with quantitative research and data [6, 8, 9].

Findings derived from qualitative research are increasingly acknowledged as important not only to accompany and support quantitative research findings to inform questions of practice and policy, but also to answer questions in their own right [10]. Qualitative systematic reviews aim to maximise the understanding of a wide range of healthcare issues that cannot be measured quantitatively; for example, they can inform understandings of how individuals and communities perceive health, manage their health and make decisions related to health service usage. Furthermore, syntheses of qualitative research can increase our understandings of the culture of communities, explore how service users experience illness and the health system, and evaluate components and activities of health services such as health promotion and community development.

Despite the increasing recognition of the importance of qualitative research to inform decision making in healthcare, historically the systematic review of qualitative research (or qualitative evidence/research synthesis) has been a highly contested topic. The debate regarding whether or not qualitative research can and should be synthesised has largely sided in support of synthesis. Despite this, there is still no international consensus to approach the combination of the findings of qualitative studies. This is evidenced by the numerous methodologies now available to incorporate and synthesise findings from qualitative research, [10] including for example meta-aggregation, meta-ethnography, realist synthesis, qualitative research synthesis and grounded theory, amongst others [5, 8, 11, 12].

Meta-aggregation has been established as a methodology for qualitative research synthesis for over a decade and was initially developed by a group led by Pearson in the early 2000s [6, 7, 13]. An underlying premise adopted by this meta-aggregative development group was that regardless of the type of evidence being synthesised, systematic reviews should be conducted in the same fashion, regardless of the type of evidence the question posed demanded. The well-established steps in the systematic review process were then tailored to qualitative evidence to develop meta-aggregation as a method of synthesis following the same principles of systematic reviews of effectiveness, whilst being sensitive to the contextual nature of qualitative research and its traditions. The meta-aggregative method has been explicitly aligned with the philosophy of pragmatism in order to deliver readily usable synthesised findings to inform decision making at the clinical or policy level [10]. As a result, the meta-aggregative approach to qualitative synthesis is particularly suited for reviewers attempting to answer a specific question about healthcare practice or summarising a range of views regarding interventions or health issues [12]. This is in contrast to other recognised approaches to qualitative synthesis, such as meta-ethnography for example, which aim to develop explanatory theories or models [12]. There are now numerous examples of meta-aggregative systematic reviews available along with detailed guidance on how to conduct this type of systematic review [6–8, 14, 15].

There have been many methodological developments aimed at improving the conduct and reporting of systematic reviews since they were first introduced. Recently, multiple international organisations that conduct systematic reviews have endorsed the recommendations from the GRADE (Grading of Recommendations Assessment, Development and Evaluation) working group. The GRADE working group has developed a systematic process to establish and present the confidence in the synthesised results of quantitative research through considering issues related to study design, risk of bias, publication bias, inconsistency, indirectness, imprecision of evidence, effect sizes, dose–response relationships and confounders of findings [16]. The outcome of this approach is a GRADE score, labelled as High, Moderate, Low or Very Low, which represents the level of confidence in the synthesised findings. This score is then applied to the major results of a quantitative systematic review. Key findings and important supporting information are presented in a ‘Summary of Findings’ table (or evidence profile). The ‘Summary of Findings’ table has been shown to improve the understanding and the accessibility of results of systematic reviews [17–19].

The GRADE approach has been widely adopted by international organisations in the conduct of quantitative systematic reviews. However, to date, there has been no widely accepted approach to assist health care professionals and policy makers in establishing confidence in the synthesised findings of qualitative systematic reviews. In light of these developments to the quantitative systematic review process, at the beginning of 2013 a methodological group was formed to consider the meta-aggregative review process specifically with the directive to detail means of establishing confidence in the findings of qualitative systematic reviews and presentation of a Summary of Findings table. The results of these discussions and the newly proposed methodology for meta-aggregative systematic reviews are presented here for consideration and promotion of further debate.

### Aim

The aim of the methodological group was twofold. Firstly, to investigate whether a system to assess the confidence in synthesised qualitative research findings using meta-aggregation could be established. Secondly, to determine and develop explicit guidance for a Summary of Findings table of qualitative systematic reviews undertaken using a meta-aggregative approach.

## Methods

The working group established comprised researchers from the Joanna Briggs Institute in Adelaide, Australia, all with experience in conducting meta-aggregative reviews. Two of the authors of this paper were involved in the development of meta-aggregation as a method for qualitative research synthesis (AP and KP). Two of the authors have also been involved with the Cochrane Qualitative Research Methods group (AP and CL).

The working group met monthly to discuss, define and determine what confidence means in terms of synthesised qualitative findings, to create a format for a ‘Summary of Findings’ table including the degree of confidence for a qualitative synthesised finding and to test and refine the newly developed methods. Consensus was reached through discussion and testing. A Delphi-like process was initiated to further refine the tool. In August 2013 the newly proposed methodology was presented to the international members of the Scientific Committee of the Joanna Briggs Institute for further consideration, discussion and ultimately, approval. Following this, it was ratified at an Institute board meeting. In October 2013 the methodology was presented in two workshops during the Joanna Briggs Institute Convention. These workshops allowed international colleagues an opportunity to provide critique and feedback on the process that had been devised. In addition, the methodology was presented to the Joanna Briggs Institute Committee of Directors, comprising over 90 international experts in research synthesis from over 20 countries for further discussion and feedback. Many of the attendees were well-versed in qualitative research synthesis and particularly the meta-aggregative approach, and were therefore seen as the ideal audience to provide feedback and critique on the methodological development of the tool. A cyclic process of feedback and review was used at all stages of the development process. The proposed methodology has been labelled ‘ConQual’. With this process now completed the group believes the methodology requires publishing for further feedback and critique from the international community of reviewers.

## Results and discussion

Meta-aggregation is a pragmatic approach to synthesis and therefore is ‘interested in how practical and useful the findings are’ [14] (p.1030). One way to improve the usefulness of the findings of a qualitative systematic review is to undertake a process to establish the confidence (defined as the belief, or trust, that a person can place in the results of the research) of these findings. Establishing confidence is of particular interest when conducting meta-aggregative synthesis as the output of this type of synthesis is ideally ‘lines of action’, which can be considered on an individual and a community level [10]. Furthermore, being explicit regarding the believability and trustworthiness of findings should be viewed as essential information for policymakers and others when considering any research findings to inform decisions in healthcare.

The working group began by considering the factors that increase or decrease the confidence in the synthesised findings of a qualitative synthesis. In the GRADE approach for quantitative research, these factors are the risk of bias, publication bias, inconsistency, indirectness, imprecision of evidence, effect size, dose–response relationships and confounders of findings [16].

After extensive debate it was agreed that there were two main elements that increased the confidence in the findings: their dependability and credibility as originally defined by Guba and Lincoln [20]. The group’s view of ‘confidence’ is similar (but not exact) to Guba and Lincoln’s ‘truth value’ of the findings of a particular inquiry [20]. The group defined ‘confidence’ as the belief, or trust, that a person can place in the results of research. Although Guba and Lincoln [20] mention other concepts (such as transferability and confirmability), it was the view of the group that these did not explicitly align with their notion of ‘confidence (i.e. truth value)’, and were more aligned to the concepts of ‘applicability’ and ‘neutrality’. The meta-aggregative approach currently incorporates methods to assess dependability and credibility and therefore there was an added practicality when deciding upon these two elements. It is worth noting however, that in the appraisal of the dependability of the research, issues of confirmability are also addressed and these are discussed further below.

The concepts of ‘dependability’ and ‘credibility’ are analogous with the ideas of ‘reliability’ and ‘internal validity’ in quantitative research. Credibility evaluates whether there is a ‘fit’ between the author’s interpretation and the original source data [21]. The concept of dependability is aligned with that of reliability in the rationalist paradigm, [20] and ‘implies trackable variability, that is, variability that can be ascribed to identified sources’ [22]. Dependability can be established if the research process is logical (i.e. are the methods suitable to answer the research question, and are they in line with the chosen methodology), traceable and clearly documented.

Determining dependability and credibility was the next challenge. The critical appraisal of qualitative research in the meta-aggregative review process was viewed as a way to assist in assessing dependability. Debate and differences of opinion continue to exist regarding the virtue of critical appraisal, or evaluation of the methodological quality of qualitative studies [6, 23, 24]. Amongst the many different methodologies that exist for the synthesis of qualitative findings, some demand critical appraisal whilst others do not [6, 25, 26].

In the meta-aggregative approach outlined by the Joanna Briggs Institute, critical appraisal is regarded as a pivotal part of the qualitative systematic review process. Critical appraisal can inform reviewers on which studies to include and can establish the quality and congruency of findings in included studies that may be used to inform healthcare practice [8]. In meta-aggregation the resultant synthesis is directly linked to all included studies. Therefore, the critical appraisal of primary studies and their subsequent inclusion or exclusion directly impacts the quality of the meta-synthesis [14].

In the meta-aggregative approach all studies included in the review are subject to a process of critical appraisal using the JBI- Qualitative Assessment and Review Instrument (JBI-QARI). This tool has been evaluated with two other critical appraisal tools for qualitative research in a comparative analysis with the authors concluding that the JBI-QARI tool was the most coherent [27].

Five questions of this checklist [27] were viewed as specifically relating to the concept of dependability. These are:

1. Is there congruity between the research methodology and the research question or objectives?
2. Is there congruity between the research methodology and the methods used to collect data?
3. Is there congruity between the research methodology and the representation and analysis of data?
4. Is there a statement locating the researcher culturally or theoretically?
5. Is the influence of the researcher on the research, and vice-versa, addressed?

It is proposed that dependability of the qualitative research study can be established by assessing the studies in the review using the above criteria. While it is not explicitly stated that ‘confirmability’ is being assessed, in addition to credibility and dependability, these five questions also address issues of confirmability, clearly encompassing ‘reasons for formulating the study in a particular way, and implicit assumptions, biases, or prejudices’ [20] (p. 248).

The next challenge was to determine an appropriate way to establish credibility. Unlike the focus of critical appraisal commonly performed as part of the systematic review process, when assessing credibility of the findings, the focus was not on the full research undertaking, but more importantly shifted to the results of the authors interpretive analysis, more commonly referred to as ‘findings’ in the literature [9, 28]. Although various definitions exist for what a finding is in qualitative research, in meta-aggregation, findings are defined as a verbatim extract of the author’s analytic interpretation accompanied by a participant voice, fieldwork observations or other data. The credibility of the finding can be established by assessing the congruency between the author’s interpretation and the supporting data. Each finding extracted from a research report can therefore be evaluated with a level of credibility based on the following ranking scale:

- **Unequivocal** (findings accompanied by an illustration that is beyond reasonable doubt and; therefore not open to challenge).
- **Equivocal** (findings accompanied by an illustration lacking clear association with it and therefore open to challenge).
- **Unsupported** (findings are not supported by the data).

By following these steps a system to establish the dependability and credibility of an individual finding is possible. However, this approach does not provide an overall ranking for the final synthesised finding. The group then returned to the principles of GRADE to determine how an overall ranking might be addressed. Within GRADE, studies are given a pre-ranking of high (for randomised controlled trials) or low (for observational studies). It was the view of the group that distinguishing between different qualitative study designs, for example, a phenomenological study or an ethnographic study, via a hierarchy was not appropriate for qualitative studies; therefore, the it was decided that all qualitative research studies start with a ranking of ‘high’ on a scale of High, Moderate, Low to Very Low.

**Figure 1:**
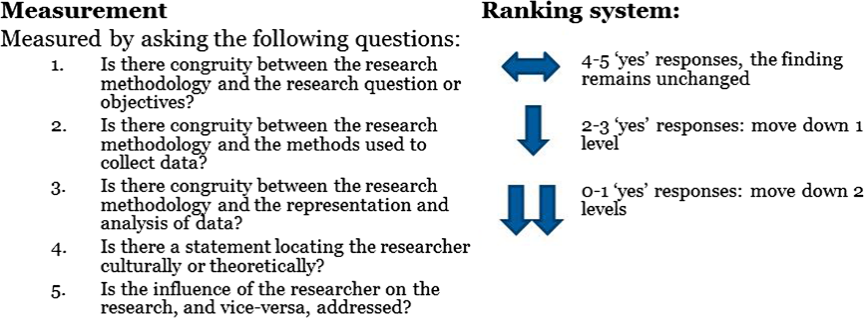
**Ranking for dependability.** This figure represents how a score for dependability is developed during the ConQual process, and is based on the response to 5 critical appraisal questions.

This ranking system then allows the findings of individual studies to be downgraded based on their dependability and credibility. Downgrading for dependability may occur when the five criteria for dependability are not met across the included studies (Figure 1). Where four to five of the responses to these questions are yes for an individual finding, then the finding will remain at its current level. If two to three of these responses are yes, it will move down one level (i.e. from High to Moderate). If zero to one of these responses are yes, it will move down two levels (from High to Low, or Moderate to Very Low). The synthesised finding may then be downgraded based on the aggregate level of dependability from across the included findings. For example, if the majority of individual findings have a ‘low’ level of dependability, this designation should then apply to the resultant synthesised finding.

**Figure 2:**
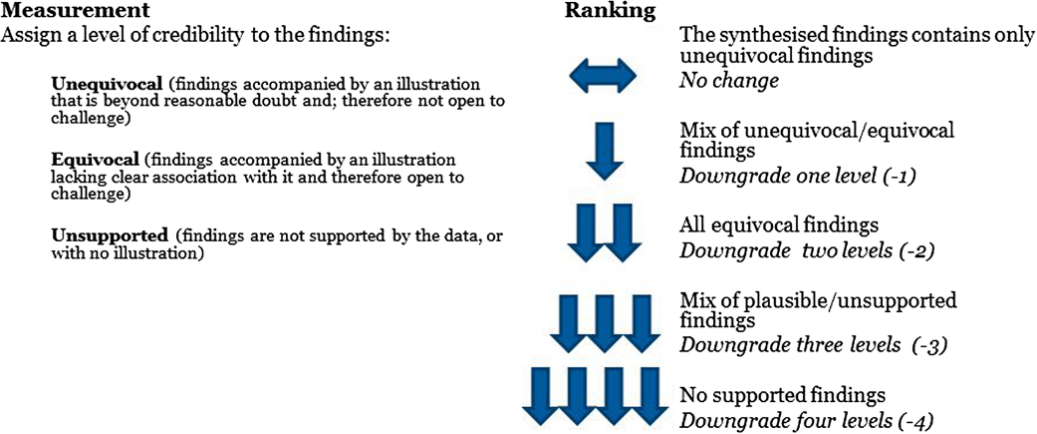
**Ranking for credibility.** This figure represents how a score for credibility is developed during the ConQual process, and is based on the congruency of the authors interpretation and the supporting data.

Downgrading for credibility may occur when not all the findings included in a synthesised finding are considered unequivocal (Figure 2). For a mix of unequivocal/equivocal findings, the synthesised finding can be downgraded one (-1). For equivocal findings, the synthesised finding can be downgraded two (-2). For equivocal/unsupported findings, it can be downgraded three (-3), and for not-supported findings, it can be downgraded four (-4).

The proposed system would then give an overall score of High, Moderate, Low or Very Low. This ranking can be considered a rating of confidence in the qualitative synthesised finding, which we have called ‘ConQual’.

The second aim of the working party was to consider the use of a Summary of Findings table. It was agreed the Summary of Findings table would incorporate the key findings of the review along with the ConQual score. The Summary of Findings table includes the major elements of the review and details how the ConQual score is developed (Table 1). The aim of the group was to create a format for the Summary of Findings table that aligned with the structure utilised by GRADE for effectiveness reviews, while presenting all of the necessary information that a reader or policy maker would find useful. Therefore, included in the table is the title, population, phenomena of interest and context for the specific review. Each synthesised finding from the review is then presented along with the type of research informing it, a score for dependability, credibility and the overall ConQual score. The type of research column (i.e. qualitative) has been included to stress to users who are more familiar with quantitative research that this is coming from a different source. Additionally, there may be scope to apply this method to synthesised findings of other types of research, including text and opinion and discourse analysis. The Summary of Findings table has been developed to clearly convey the key findings to a reader of the review in a tabular format, with the aim of improving the accessibility and usefulness of the systematic review. This system has been trialled by the working party with a number of systematic reviews, with one example illustrated below in Table 1.

**Table 1:** **ConQual summary of findings example**

Systematic review title: the patient experience of high technology medical imaging: a systematic review of the qualitative evidence				
Population: persons who had undergone high technology medical imaging				
Phenomena of interest: the meaningfulness of a patients experience of undergoing diagnostic imaging using high technology				
Context: male and female adult patients presenting to a medical imaging department				
**Synthesised finding**	**Type of research**	**Dependability**	**Credibility**	**ConQual score**
People undergoing imaging often expect a health issue to be found during their scan, which can then	Qualitative	Downgrade 1 level*	Downgrade 1 level**	**Low**

* Downgraded one level due to common dependability issues across the included primary studies (the majority of studies had no statement locating the researcher and no acknowledgement of their influence on the research).

** Downgraded one level due to a mix of unequivocal and equivocal findings.

There is a tool similar to ConQual currently in development called CerQual. The focus of this tool is to establish how much certainty (or confidence) to place in findings from qualitative evidence syntheses [29–31]. Certainty is described as ‘how likely it is that the review finding happened in the contexts of the included studies and could happen elsewhere’ [30]. The elements that contribute to the overall degree of certainty are the methodological quality of the study (in one review protocol this is determined by the Critical Appraisal Skills Programme quality assessment tool) [30] and the plausibility (or coherence, established when authors are able to ‘identify a clear pattern across the data contributed by each of the individual studies’) [31] of the review finding. The methodological quality of qualitative studies is linked to their dependability (as mentioned in Glenton’s protocol), which is similar to the ConQual approach. The two tools (ConQual and CerQual) share similar characteristics in the following ways; they both aim to provide a qualitative equivalent to the GRADE approach, they both propose a final ranking, and they both assess methodological quality or dependability. However, the main point of difference is that ConQual focuses on the credibility of the findings whereas CerQual focuses on the plausibility (or coherence) [32] of the findings. Due to this difference it is reasonable to suggest that both tools can be considered during the conduct of qualitative research synthesis, with ConQual particularly suited to meta-aggregative reviews. At the time of development and reporting of the ConQual system, we were not aware of any testing being conducted using the CerQual approach for meta-aggregative reviews or the ConQual approach for other types of qualitative research synthesis. In these early stages of development it is difficult to provide concrete guidance on when to choose either the ConQual or CerQual approach. Over time it may emerge more clearly when one tool should be used in preference to the other based on their relative merits. Those researchers conducting qualitative research syntheses should carefully consider which tool will best suit their purpose.

As is to be expected with new methodologies there are some limitations to the ConQual approach. The methods detailed within this paper were developed specifically for qualitative synthesis using meta-aggregation. In principle, other qualitative research synthesis methodologies could adopt this approach (perhaps with some slight modifications), as a ConQual ranking can be generated with any approach where findings are identifiable and the credibility of findings and dependability of research are able to be assessed. As with any new methodological developments there will almost certainly be opposing views. Potential criticism of the ConQual approach may exist around the use of an ordinal scale for ranking the confidence of qualitative research. However, we argue that this approach is not only appropriate, but above all practical. The process is a further step towards assisting policymakers and healthcare professionals in incorporating the evidence into healthcare related policy and decisions. The movement towards developing and establishing confidence in the synthesised findings of qualitative systematic reviews is a relatively new concept. It is hoped that this paper will stimulate discussion thereby improving and continuing development within this area.

## Conclusions

The explicit aim of meta-aggregative reviews is to ensure that the final synthesised finding can be used as a basis to make recommendations for healthcare practice or inform policy. It is believed that the development and use of the ConQual approach will enable users of qualitative systematic reviews to establish confidence in the evidence produced in these types of reviews and serve as a practical tool to assist in decision making.
